# CT perfusion as a useful tool in the evaluation of leuko-araiosis

**DOI:** 10.2349/biij.2.2.e16

**Published:** 2006-04-01

**Authors:** N Ramli, KL Ho, O Nawawi, HT Chong, CT Tan

**Affiliations:** Departments of Biomedical Imaging and Medicine, University Malaya Medical Centre, Kuala Lumpur, Malaysia

**Keywords:** CT perfusion, chronic ischemia, dementia, leuko-araiosis

## Abstract

**Background:**

Leuko-araiosis (LA) and dementia are common geriatric conditions but their pathogenesis and clinical significance are not completely understood. An evaluation of CT perfusion (CTP) in both these conditions can further enhance the understanding of these diseases.

**Methods:**

Twenty-one patients with LA and 21 age-matched controls were studied with CTP and assessed for their cognitive function. The subjects were classified into four groups: Group 1, with LA (*n* = 21); Group 2, without LA (*n* = 21); Group 3, with dementia (*n* = 7); Group 4, without dementia (*n* = 11). The mean cerebral blood flow (CBF), cerebral blood volume (CBV) and mean transit time (MTT) values were compared between groups 1 and 2, while mean CBF values were compared between groups 3 and 4.

**Results:**

Mean white matter CBF was considerably reduced in patients with LA in the frontal region by 42% (*p* = 0.000), basal ganglia by 37% (*p* = 0.000) and occipital region by 18% (*p* = 0.019). The mean white matter CBV was reduced in patients with LA in the frontal region by 36% (*p* = 0.000) and basal ganglia by 28% (*p* = 0.017). The mean white matter CBF was dramatically reduced in patients with dementia in the frontal region by 44% (*p* = 0.000), basal ganglia by 32% (*p* = 0.038) and occipital regions by 24% (*p* = 0.001).

**Conclusion:**

The CTP showed reduced white matter CBF and CBV in patients with LA. This is consistent with chronic ischemia as the pathogenesis of LA. The CTP is also a potentially important technique in the diagnosis and management of dementia, because of its ability to reveal cerebral hypoperfusion.

## INTRODUCTION

Leuko-araiosis (LA) is the term introduced by Hachinski *et al* to designate periventricular or subcortical (centrum semiovale) areas of hypodensity on computed tomography (CT) or hyperintensity on T_2_-weighted magnetic resonance imaging (MRI). LA is a common condition, with epidemiological studies demonstrating a high prevalence in subjects over 65 years of age, as evaluated by CT or MRI. It is probably caused by chronic cerebral ischemia but the pathogenesis and its clinical significance are not completely understood. Some individuals remain asymptomatic for prolonged periods, while others develop gait disturbance, cognitive impairment, mood disorders, disability, and even dementia. LA increases overall morbidity and mortality and also the risk of stroke. Further understanding of the pathogenesis of LA is essential because it is potentially preventable and modifiable [1,2].

Dementia is a highly widespread and morbid condition frequently found in the elderly, especially those affected by LA. Clinical identification and diagnosis of dementia or predementia is especially difficult in the early stages. However, the early and accurate diagnosis is important because early treatment is now possible. Functional brain imaging techniques such as PET and SPECT can provide substantial assistance in the initial diagnosis of dementia, but are limited because of factors such as low availability and high costs [3,4,5].

Recent developments in helical and high-speed continuous data technology have enabled CTP using iodinated contrast material. CTP of the brain is a proven tool, especially in acute stroke and oncology cases [6,7]. With the evolution of spiral multi-slice CT technology and its increasing availability, CTP can play an important role in clinical techniques. Our study examined the correlation of cerebral perfusion with LA and dementia, to prove that the pathogenesis of the LA is chronic cerebral ischaemia. We also tested the hypotheses, that the mean cerebral blood flow (CBF) in the cerebral white matter in patients with LA is lower than in patients without, within the same age group; and that the mean CBF in the cerebral white matter of patients with dementia is lower than in patients without.

## MATERIALS AND METHODOLOGY

### Subjects

**Figure 1:**
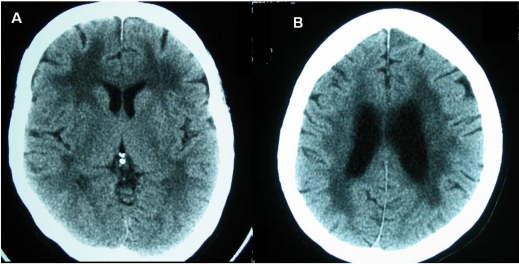
Plain CT brain at the basal ganglia (A) and corona radiata (B) levels showing periventricular white matter lucency in a patient with leuko-araiosis.

The subjects were selected from a group of patients who were referred to our department for CT examination of the brain. The reasons for referral were stroke (*n* = 18), dementia or cognitive impairment (*n* = 14), headache (*n* = 6), giddiness (*n* = 5), psychiatric disorder (*n* = 4), syncope (*n* = 1) and metastatic malignancy of the brain (*n* = 1). The subjects were selected based on the identification of LA in non-contrasted CT brain using standard window (Figure 1). A total of 23 patients with LA were identified and they underwent CTP on the same day as the scam. A total of 26 patients without LA were also selected for CTP as an age-matched control sample. All subjects were assessed with Mini Mental State Examination (MMSE) either before or after the procedure. Patients with an MMSE score of less than 24 and with clinical features of dementia or cognitive impairment were used for the sample with dementia. The following information was collected from the patients: age, sex and risk factors (diabetes mellitus, hypertension and cardiovascular disease). Based on the non-contrasted CT findings and MMSE scores, the subjects were classified into four groups: Group 1, patients with LA (*n* = 21, 10 Male, 11 Female); Group 2, patients without LA (*n* = 21, 9 Male, 12 Female); Group 3, patients with dementia (*n* = 7, 3 Male, 4 Female; 3 with LA, 4 without LA); Group 4, patients without dementia (*n* = 11, 6 Male, 5 Female; all with no LA).

The clearance from the Ethical Committee was obtained prior to the commencement of this study, along with informed consent from the subjects or their next of kin. Those patients with contraindication to contrast agents, history of allergy, renal impairment, or with intracranial bleed or massive mass effect on non-contrasted CT brain were excluded from the study.

### Scanning Technique

All CT examinations were performed on a 16 slice multidetector CT scanner (GE Lightspeed; GE Medical Systems, Milwaukee, WI, USA). The non-contrasted CT examinations were done using either 3 or 5 mm transverse sections through the posterior fossa and 5 or 10 mm transverse sections through the supratentorial region.

For the CTP protocol, continuous scanning of four adjacent 5-mm axial sections was performed with a 45-s cine with a 1-s interval, retrospectively reconstructed to a 0.5-s interval, and acquired at 80 kVp using 190 mAs to 200 mAs. For this examination a bolus of 40 ml contrast material (Ultravist, Schering, Berlin) injected at 4 ml s^-1^ was used. A 25-cm field of view was used for all the scans, which were reconstructed with a matrix of 512 x 512 pixels. The scans were obtained in the transverse plane, and the examination covered the common areas of LA, which are the basal ganglia and the corona radiata.

### Analysis of Scans

Two radiologists independently reviewed the non-contrasted CT scans on an imaging workstation (Advantage Windows; GE Medical Systems, Milwaukee, Wisconsin). The presence of LA and other lesions such as focal infarcts were documented. The CTP scans were analyzed by two radiologists using the same imaging workstation, with commercial CTP analysis software (CTP; GE Medical Systems, Milwaukee, Wisconsin), to create maps of CBF, CBV and mean transit time (MTT). The more cephalad section of the 10 mm sections (x 2) or 5 mm sections (x 4) were analyzed to maintain a uniform number of sections in all subjects.

Both the radiologists placed ROI (3–7 mm^2^) on the input arteries and veins at the time of evaluation of the scan by using the same protocol, for which contrast-enhancement curves were generated. For most patients, the larger of the two anterior cerebral arteries was chosen for placement of the ROI, providing the arterial input function, and the superior sagittal sinus was chosen for placement of the ROI, providing the venous outflow function. These vessels were chosen because they can be identified in most subjects and also because their courses run almost perpendicular to the transverse plane of the section used for the CT scanning of the brain. By choosing such vessels errors due to volume-averaging artifacts would be decreased.

**Figure 2:**
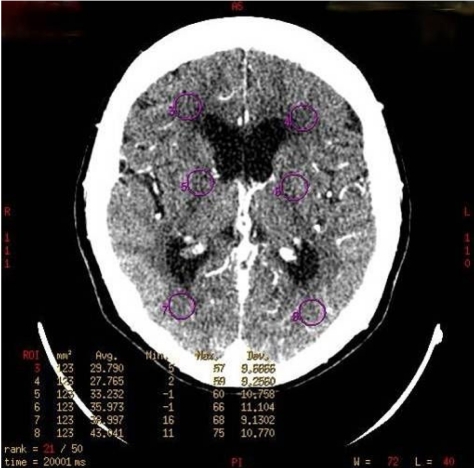
Axial CTP scan showing six circular ROI (in purple) placed at both frontal, basal ganglia and occipital regions, indicating location of CTP measurement.

For the measurement of the brain parenchyma perfusion parameters (CBF, CBV, MTT), six small circular ROI were drawn on the white matter regions of frontal, basal ganglia and occipital lobes. The size of the ROI was standardized in all samples, at 120–125 mm2 (Figure 2). The specifications were because an ROI of this size is technically easy to create and highly reproducible during the post-processing time, and is optimal for inclusion and coverage of most representative areas showing white matter changes in LA.

**Figure 3:**
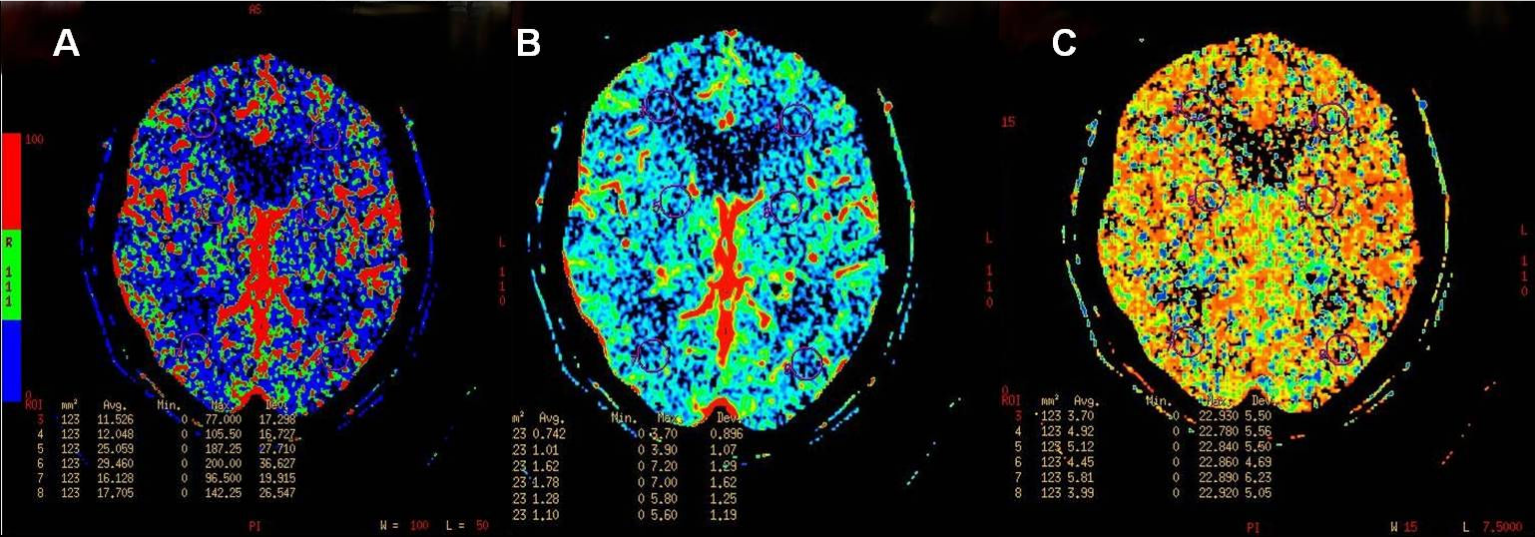
CBF (A), CBV (B) and MTT (C) maps of a patient with leuko-araiosis.

Next, the perfusion maps were created in the following order: CBF, CBV and MTT (Figure 3), following which the mean value of that perfusion parameter contained within each of the six ROI was recorded. These mean values were compared between groups 1 and 2, and groups 3 and 4. For statistical analysis we used the Mann-Whitney test, with a statistical significance level set at *p*<0.05.

## RESULTS

### Subjects

A total of 49 patients underwent plain CT brain scan, CTA and CTP in this study. There were 23 subjects in the LA group and 26 in the control group. The study population comprised 27 females (55.1%) and 22 males (44.9%). The mean age for study population was 70.1 years (70.19 in LA group; 69.86 in non-LA; *p*-value>0.05).

The risk factor profile for patients with and without LA is shown in Table 1 Patients with LA showed higher prevalence of hypertension, diabetes mellitus, cardiovascular disease and cerebrovascular accidents. The mean MMSE score was also lower in the LA group (23.53), as compared to the control group (27.8), but this is not a considerable statistical difference (*p*-value>0.05).

**Table 1:** Risk factors profile for patients with or without leukoaraiosis

**Characteristic**	**LA( n = 23 )**	**Non-LA( n = 26 )**
Age, mean	70.19	69.86
Age, range	51-87	51-80
Female	12 (52.2%)	15 (57.7%)
Male	11 (47.8%)	11 (40.7%)
Hypertension	15 (65.2%)	9 (34.6%)
Diabetes mellitus	9 (39.4%)	7 (26.9%)
CVD	8 (34.8%)	2 (7.7%)
CVA	17 (73.9%)	9 (34.6%)
Mean MMSE	23.53 / 30	27.83 / 30

CVD=cardiovascular disease

CVA=cerebrovascular accident

### CT Perfusion

CTP was successfully performed in 85.7% (*n* = 42; LA 21, Non-LA = 21) of the cases. However, it was unsuccessful in seven cases (LA 2, non LA 5), because of motion artifacts and technical errors encountered during the procedure.

We found no dramatic difference between the right and left hemisphere CTP parameter values, and therefore the values of the regions of interest from both sides were pooled. Mean white matter values of frontal, basal ganglia and occipital regions were obtained by averaging values from both sides of these regions.

### (a) Comparison of mean perfusion parameter values between LA (Group1) and non-LA (Group 2)

There was a considerable difference between of the mean white matter CBF between groups 1 and 2. In Group 1, it was dramatically reduced in patients with LA in the frontal by 42% (*p* = 0.000), basal ganglia by 37% (*p* = 0.000), and occipital regions by 18% (*p* = 0.019), as compared with Group 2 (Table 2).

**Table 2:** Mean CBF values (ml/min/100g), Mean CBV values (ml/100g) and MTT (sec) in the leukoaraiosis and the non- leukoaraiosis groups

**Region**	**CBF**		**CBV**		**MTT**	
	LA	Non LA	LA	Non LA	LA	Non LA
Mean frontal WM	10.61	18.47	0.87	1.38	5.59	5.05
	*p*=0.000		*p*=0.000		NS	
Mean basal ganglia WM	20.33	32.47	1.42	1.99	4.98	4.29
	*p*=0.000		*p*=0.017		NS	
Mean occipital WM	15.31	18.74	1.25	1.41	5.72	5.30
	*p*=0.019		NS		NS	

WM = white matter, NS = Not Significant

The mean white matter CBV was also considerably reduced in patients with LA in the frontal region by 36% (*p* = 0.000) and the basal ganglia by 28% (*p* = 0.017), but not in the occipital region (*p* = 0.289). As for the mean MTT values, there was no significant difference between groups 1 and 2 for all the regions measured.

### (b) Comparison of mean perfusion parameter values between dementia (group 3) and non-dementia (group 4)

When compared with Group 4, the mean white matter CBF of the patients in Group 3 were greatly reduced in the frontal region by 44% (*p* = 0.000), basal ganglia by 32% (*p* = 0.038) and occipital region by 24% (*p* = 0.001). The mean white CBV of patients with dementia were also greatly reduced in the frontal region by 40% (*p* = 0.011) compared with the control group. The mean white matter MTT values were also not considerably different between these two groups (Table 3).

**Table 3:** Mean CBF values ( ml/min/100g), Mean CBV values (ml/100g) and MTT (sec) in dementia and non-dementia patients

**Region**	**CBF**		**CBV**		**MTT**	
	Dementia	Non- dementia	Dementia	Non- dementia	Dementia	Non- dementia
Mean frontal WM	11.90	21.26	12.93	22.05	20.07	17.50
	*p*=0.000		*p*=0.011		NS	
Mean basal ganglia WM	24.32	36.03	16.00	20.09	20.07	17.50
	*p*=0.038		NS		NS	
Mean occipital WM	15.47	20.56	13.79	21.50	19.32	17.98
	*p*=0.001		NS		NS	

WM = white matter , NS = not significant

## DISCUSSION

The nature, pathogenesis and clinical significance of LA are not well understood. Many studies have emphasized the significant correlation of this condition with aging, hypertension, diabetes mellitus, cardiovascular disease, cerebrovascular accident and cognitive impairment [1,2,8-11]. Our study revealed similar results. LA is mainly seen in the elderly in this study, with a mean age of 70.1 years, compared to 73.7 years in a study by Wiszniewska *et al* [9]. Higher prevalence of hypertension, diabetes mellitus, cardiovascular disease and cerebrovascular accident are noted in the LA group as compared with the control group. Aging, hypertension and diabetes mellitus induce arteriosclerotic changes on the small penetrating arteries and arterioles of white matter, suggesting that LA is primarily a small vessels disease. The correlation between LA and cognitive impairment is well established [12-14] and consistent results are observed in this study, where the mean MMSE score in the LA group is lower than in the control group.

In the past, many researchers have attempted to prove chronic ischemia as the pathogenesis of this entity using various imaging modalities. Markus *et al* had shown reduced cerebral blood flow in white matter in LA using quantitative exogenous contrast-based perfusion MRI [15]. By using single photon emission computed tomography, Starkstein *et al* found that patients with Alzheimer’s disease, and LA had significant lower bilateral perfusion in the basal ganglia, thalamus, and frontal lobes than in Alzheimer patients without LA [16]. Miyazawa *et al* demonstrated reduced CBF in centrum semiovale of asymptomatic individuals with LA, using xenon-contrasted CT. Their study also demonstrated that the CBF values were much reduced in cases with more severe white matter changes [17]. CTP is a new imaging method that allows rapid qualitative and quantitative evaluation of cerebral perfusion, which has not been used in the evaluation of LA.

We have shown significant reduction of white matter CBF and CBV in patients with LA. In chronic ischemia, reduction in CBF is associated with reduction in CBV due to the loss of the autoregulatory mechanism of the involved vessels, in contrast to acute ischemia where the CBV is maintained or elevated due to an intact autoregulatory function. Prolongation of MTT is always noted in cerebral infarction due to vascular obstruction or sluggish flow [6]. No significant prolongation of MTT was noted in this study sample, possibly due to the establishment of effective collateral pathways in chronic ischemia that has shortened the transit time of blood flow. Overall, these results provide further evidence for the role of chronic ischemia in the pathogenesis of LA. The absolute values of CBF obtained in the LA group were similar to those in previous studies [15,16]. The mean frontal white matter CBF value in our LA group were 10.61 ml/min/100g, compared with 13.65 ml/min/100g measured by Markus, with perfusion MRI [15]. Miyazawa *et al* showed mean centrum semiovale CBF values ranging from 11.31-24.27 ml/min/100g [17] while our study obtained values of 10.61-20.33 ml/min/100g. These findings suggest the validity of the CBF measurement using CTP.

Our study also showed a considerable reduction of CBF in all regions, in Group 3. A correlation between cognitive ability and cerebral perfusion had been demonstrated using xenon-contrasted CT, PET and SPECT in previous studies [18-20]. Meyer *et al* used xenon-contrasted CT and found that reduced mean Cognitive Capacity Screening examination (CCSE) scores correlated directly with CBF reductions in patients with multi-infarct dementia and dementia of the Alzheimer’s type [18]. Terayama *et al*, also using xenon-contrasted CT, showed that the perfusion of cerebral white matter was diffusely and severely reduced in the frontal, temporal and occipital regions of 12 patients with dementia [19]. Few authors have also found that SPECT is able to reveal a specific pattern of cerebral perfusion defects, depending upon the type of dementia, allowing for specific diagnosis of dementia [20,21]. Our study has shown that CTP is a valid and useful imaging modality with the ability to demonstrate cerebral perfusion changes in patients with dementia. Thus, coupled with clinical assessment, CTP should potentially enhance the diagnostic accuracy of dementia. However, our study has not been designed to analyze CTP among the various types of dementia, and clinical grading of dementia from mild to severe is expected to affect the CTP data. Further study with a larger sample of patients with dementia, and a study design that allows differentiation of various types of dementia is warranted. This can further investigate the feasibility of CTP as a useful imaging method in diagnosing specific types of dementia, which had been shown possible with SPECT.

## CONCLUSION

CTP is a non-invasive, fast, easy-to-perform imaging tool, which is valid and comparable with other imaging modalities in measuring cerebral perfusion in patients with LA and dementia. By using CTP, we have shown significant reduced white matter CBF and CBV in patients with LA. This is consistent with chronic ischemia as the pathogenesis of LA. We have also demonstrated that CTP is a potentially important clinical imaging technique in the diagnosis and management of dementia, in view of its ability to show significant cerebral hypoperfusion in these patients.
